# Reduction in terminally differentiated T cells in virologically controlled HIV-infected aging patients on long-term antiretroviral therapy

**DOI:** 10.1371/journal.pone.0199101

**Published:** 2018-06-13

**Authors:** Nicole E. Behrens, Anne Wertheimer, Stephen A. Klotz, Nafees Ahmad

**Affiliations:** 1 Department of Immunobiology, College of Medicine, University of Arizona, Tucson, Arizona, United States of America; 2 Department of Medicine, College of Medicine, University of Arizona, Tucson, Arizona, United States of America; 3 Bio5 Institute, University of Arizona, Tucson, Arizona, United States of America; University of Texas Medical Branch at Galveston, UNITED STATES

## Abstract

Several studies have shown an increased accumulation of terminally differentiated T cells during HIV infection, suggestive of exhaustion/senescence, causing dysregulation of T cell homeostasis and function and rapid HIV disease progression. We have investigated whether long-term antiretroviral therapy (ART), which controls viremia and restores CD4 T cell counts, is correlated with reduction in terminally differentiated T cells, improved ratios of naïve to memory and function of T cells in 100 virologically controlled HIV-infected patients. We show that while the median frequencies of terminally differentiated CD4^+^ and CD8^+^ T cells (CD28^-^, CD27^-^, CD57^+^ and CD28^-^CD57^+^), were higher in the virologically controlled HIV-infected patients’ cohort compared with uninfected individuals’ cohort, the frequencies of these cells significantly decreased with increasing CD4 T cell counts in HIV-infected patients. Although, the naïve CD4^+^ and CD8^+^ T cells were lower in HIV patients’ cohort than uninfected cohort, there was a significant increase in both naïve CD4^+^ and CD8^+^ T cells with increasing CD4 T cell counts in HIV-infected patients. The underlying mechanism behind this increased naïve CD4^+^ and CD8^+^ T cells in HIV-infected patients was due to an increase in recent thymic emigrants, CD4^+^CD31^+^, as compared to CD4^+^CD31^-^. The CD4^+^ T cells of HIV-infected patients produced cytokines, including IL-2, IL-10 and IFN-γ comparable to uninfected individuals. In conclusion, virologically controlled HIV-infected patients on long-term ART show a significant reduction in terminally differentiated T cells, suggestive of decreased exhaustion/senescence, and improvement in the ratios of naïve to memory and function of T cells.

## Introduction

Human immunodeficiency virus (HIV) infection increases the population of terminally differentiated T cells, termed as premature aging of T cells [1–3], and rapid HIV diseases progression in infected patients with uncontrolled viremia [1–3]. Several HIV-induced immunologic changes in T cells are also seen in uninfected elderly population, referred as immunosenescence [1, 3], which likely occurs due to continuous viral replication, extreme activation and exhaustion of CD8^+^ T cells [3–5]. These age-related changes may result in dysregulation of T cell function and homeostasis and diminish the breadth of immune response in HIV-infected older individuals, which may contribute to increased susceptibility to new infections, frequent recurrent infections, and poor response to vaccinations [4]. While long-term antiretroviral therapy (ART) has reduced the viral loads and restored CD4 T cell counts in many HIV-infected patients, it is not clear whether there is improvement in terminal differentiation, homeostasis and functions of T cells.

HIV-mediated immune dysfunctions and senescence are associated with many common immune dysregulations, such as impaired thymic function [6, 7], altered ratios of circulating naïve to memory T cells [6, 8], increased expression of CD95 on T cells [9], diminished expression of CD28 costimulatory molecule on CD8^+^ T cells [1, 6] and impaired lymphoproliferative responses to mitogens/antigens [10]. Both in HIV infection and aging, T cell homeostasis is disturbed as naïve T cells decrease compared with memory T cells and CD4^+^ T cells decline with respect to CD8^+^ T cells [6–8]. Even in some patients with reduced viral load due to ART, physiological limitations of CD4^+^ T cell renewal worsen the reconstitution of depleted memory CD4^+^ T cells because of impaired thymic output [11, 12]. As T cell homeostasis may not reach a balanced state in HIV individuals after many years of ART [13], the distribution and function of T cell subsets in HIV-infected aging patients receiving ART are not clearly defined.

Earlier studies reveal that both HIV infection and aging induce terminal differentiation of T cells [1, 2], which is likely accelerated in HIV-infected older individuals. T cell homeostasis is altered during HIV infection, first by depleting the memory CD4^+^ T cell pool and then by infecting naïve CD4^+^ T cells as well as recruiting both naïve CD4^+^ and CD8^+^ T cells into the memory pools due to chronic immune activation [14–16]. While costimulatory molecule CD28, essential for cytokine expression, proliferation and survival of T cells [17, 18], is lost in HIV infection and aging [19, 20], terminal differentiation marker CD57 on T cells, commonly associated with conditions of chronic antigenic exposure, is expressed at higher levels and inversely related to CD28 expression [1, 21]. Similar dysregulation of cytokines is seen by CD4^+^ T cells in HIV infection and aging, including reduced expression of IL-2 [22] and increased expression of IL-1β, IL-6, TNF-α, and IFN-γ [23, 24]. Several studies have shown that there is an increased accumulation of terminally differentiated CD28^-^CD57^+^ T cells in HIV-infected individuals with uncontrolled viremia and lower CD4 T cell counts, suggestive of exhausted/senescent T cells, associated with rapid HIV disease progression [1, 3, 25]. While HIV-infected patients are being successfully treated with ART and many have achieved controlled viremia and increased CD4 T cell counts, it is not clear whether there is a reduction in terminally differentiated T cells, and improvement in the ratios of naïve to memory and function of T cells.

In this paper, we show that HIV-infected patients with controlled viremia and improved CD4 T cell counts due to long-term ART have achieved a reduction in the frequencies of terminally differentiated, C28^-^CD57^+^ CD4^+^ and CD8^+^ T cells, suggestive of diminished exhaustion/senescence. We also show an improvement in the ratios of CD4^+^ to CD8^+^ T cells, increased naïve T cells, changes in the naïve T cells phenotype, and decreased memory T cell populations in these HIV-infected patients. Furthermore, the CD4^+^ T cells of these HIV-infected patients produced cytokines, including IL-2, IL-10 and IFN-γ, suggesting improved T cell function.

## Materials and methods

### Human subjects

Our study was approved by the Institutional Review Board at the University of Arizona. Informed and signed consent was obtained from all participants in the study. The HIV+ patients’ cohort consists of 100 clinically diagnosed HIV-infected individuals (median age: 53 years and CD4 T cell counts: 528 cells/mL) belonging to different ethnicities with varying ages ranging from 22–81 years (84 males and 16 females) receiving health care and antiretroviral therapy (ART) for at least 3 years and up to 30 years at the Petersen HIV Clinic Division of Infectious Disease, Banner University Medical Center, Tucson, Arizona. The uninfected individuals’ cohort (control) includes 75 healthy age-matched and different ethnicities (median age: 53 years and CD4 T cell counts: 958 cells/mL) self-reported HIV-uninfected controls (40 males and 35 females). The HIV+ patients’ cohort consisted of 12 patients below age 40 years and 88 above 40, while the uninfected controls cohort consisted of 14 individuals below age 40 and 61 above 40. The subjects’ demographics and laboratory parameters are shown in Table 1 and Table 2. Blood samples were drawn into 4 sodium heparin 10.0 mL BD Vacutainer tubes (BD, Sunnyvale, CA) or into 4 10.0 mL CPT mononuclear cell preparation sodium heparin tubes (BD, Sunnyvale, CA). The blood samples were processed at the University of Arizona Biorepository Laboratory and peripheral blood mononuclear cells (PBMC) were cryopreserved for future analysis. Briefly, PBMC and plasma were separated using either density centrifugation SepMate-50 (Stemcell Technologies Inc, Vancouver, BC) or CPT per manufacturers recommendations, and stored in cryopreservation media. We also collected one K2 EDTA tube to determine complete blood counts, using an A^c^-T 5diff CP machine (Beckman Coulter, Pasadena, CA).

**Table 1:** HIV-infected and uninfected-control cohorts’ demographics.

Cohorts	Number of Subjects (n)	Age (yrs)	Median Age (yrs)	Total Females (n)	Total Males (n)	Ethnicity
HIV-Infected Cohort	100	22–81	53	16	84	A, AA, MA, O, H, W
Control Cohort	75	23–75	53	35	40	AA, AI, H, W

A: Asian, AA: African American, AI: American Indian, MA: Mexican American, O: Other, H: Hispanic, W: White

**Table 2:** HIV-infected and uninfected-control cohorts’ laboratory parameters.

Cohorts	Lymphocyte Count (cells/ml)	CD4 Count (cells/ml)	Median CD4 Count (cells/ml)	CD8 Count (cells/ml)	Median CD8 Count (cells/ml)	Viral Load (copies/ml)
HIV-Infected Cohort	580–4180	70–1592	528	149–1680	645	UD to <100
Control Cohort	980–4420	427–1845	958	114–786	338	N/A

UD: Undetected

### Flow cytometry (FCM)

Cryopreserved PBMC (1-2x10^6^/sample) were stained with LIVE/DEAD Fixable Dead Cell Stain-AQUA (Invitrogen) and T cell markers in various combinations. The following mAbs were used to differentiate T cell subsets: CD3–BV570 (BioLegend), CD4 –APC (eBioscience), CD8β –ECD (Beckman Coulter), CD95 –BV421 (BioLegend), CD28 –PerCp/Cy5.5 (BioLegend), CCR7 –FITC (BD Pharmogen), CD45RA–BV605 (BioLegend), CD27 –PE-e610 (eBioscience), CD57 –BV570 (BioLegend), IFN-γ –APCe780 (eBioscience), IL-2 –PE-Cy7 (eBioscience), IL-10 –PE (BD Pharmogen). Additionally, in select experiments CD4 –APC Fire 750, CD57 –APC, CD27 –PE, CD31 –PE-Cy7 from BioLegend were used.

Cryopreserved PBMC were thawed and plated at 1x10^6^ viable cells/well in 5% X-VIVO 15 media (X-VIVO 15 + 5% male human AB serum) in a 96 well round bottom tissue culture plate; and rested overnight in a 37^°^C incubator with 5% CO_2_. Post overnight rest the appropriate wells were stimulated with PMA-Ionomycin (eBioscience) at a 1:500 dilution for 2 hours at 37^°^C and 5% CO_2_. All wells regardless of treatment condition incubated 2 hours with a combination of 1X Brefeldin A Solution (3.0μg/ml) and 1X Monensin Solution (2μM/ml) (eBioscience). Post incubation, cells were washed 2X with 200μl PBS and centrifuged at 1650 RPM for 4min. Cells were stained with LIVE/DEAD Aqua per manufacturers protocol for 30min at 4^°^C in dark. Cells were washed 2X with PBS and centrifuged at 1650 RPM for 4min. Cells were stained for surface markers in 50μl of Brilliant Stain Buffer (BD Bioscience) in various combinations for 30min at 4^°^C in dark. Cells were washed 2X with FACs Buffer (PBS + 2% FBS) and centrifuged at 1650 RPM for 4min. Cells are then incubated for 30min at 4^°^C in dark with Foxp3/Transcription Factor Staining Buffer set (eBioscience), for fixation and permeabilization of cells for intracellular staining. Cell are then washed 2X with Permeabilization buffer (perm buffer) and centrifuged at 1750 RPM for 4min. Cells were then stained for intracellular markers in various combinations in 50μl perm buffer for 30min at 4^°^C in dark. Cells were finally washed 3x in FACs buffer and re-suspended in 120μl of FACs buffer for analysis on the BD LSR II instrument, using DiVa acquisition (BDIS, Mountain View, CA) and the FlowJo analysis software (TreeStar Inc., Ashland, OR). All data was collected using set optimized standard voltages and calibrated using Rainbow beads (BD Bioscience) on a daily basis.

For identification of the T cell subsets, we used a comprehensive gating strategy. All samples were first gated for the lymphocyte population using side scatter and forward scatter, followed by selection for viability. Analysis for the CD4^+^ T cell population was gated through isolation of CD3^+^ followed by CD4^+^ and the CD8^+^ T cell population was gated directly using CD8β. Following differentiation of CD4^+^ and CD8^+^ T cells, central memory (CD28^Hi^CD95^Hi^CD45^Lo^CCR7^Hi^), effector memory (CD28^Lo^CD95^Hi^CCR7^Lo^) and naïve T cell populations (CD28^Int^CD95^Lo^CD45^Hi^CCR7^Hi^) populations were gated through the markers CD95 and CD28 first, then sub-gated through the markers CD45RA and CCR7. For identification of the three populations described by Cao et.al. the early (CD28^+^CD57^-^), intermediate (CD28^-^CD57^-^) and late (CD28^-^CD57^+^) stage T cell populations, we first differentiated for CD4^+^ and CD8^+^ T cells then gated though CD28^+/-^, followed by CD57^+/-^.

### Statistical analysis

Overall population totals were graphed using medians and 95% confidence intervals. An unpaired, non-parametric Mann-Whitney test was used to determine statistical significance between the HIV-uninfected control population totals and HIV-infected patient population totals. Linear regression models were used for all linear plots comparing a population to CD4 T cell counts. Statistical significance was determined by comparing the linear regression slope to a slope of zero. In the event that both linear regression slopes were significantly non-zero, the slopes were compared to each other to see if they differed significantly. Software used was Graphpad Prism 7 (LA Jolla, CA).

## Results

### Redistribution of T cell subsets in virologically controlled HIV-infected aging patients on ART

**Fig 1:**
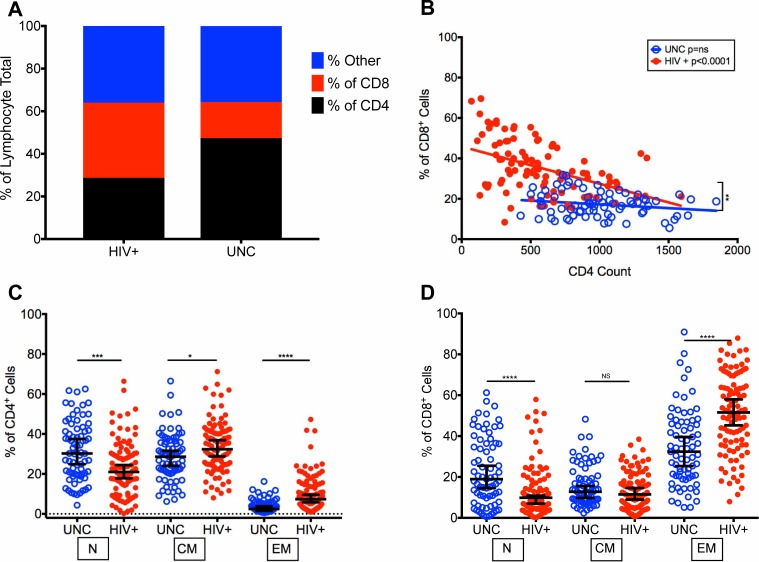
Distribution of CD4^+^ and CD8^+^ T cell subsets in HIV-infected patients with controlled viremia on long-term antiretroviral therapy (ART). (A) Lymphocyte distribution measured as frequencies of CD4^+^, CD8^+^, and non-T cell populations in HIV-infected patients (HIV+) and uninfected controls (UNC). (B) Frequencies of CD8^+^ T cells by increasing CD4 T cell counts in HIV+ and UNC. Frequencies of CD4^+^ (C) and CD8^+^ (D) T cell subsets defined as naïve (N, CD28^Int^CD95^Lo^CD45RA^Hi^CCR7^Hi^), central memory (CM, CD28^Hi^CD95^Hi^CD45RA^Lo^CCR7^Hi^) and effector memory (EM, CD28^Lo^CD95^Hi^CCR7^Lo^). (n = 100 HIV+, n = 75 UNC) (*p<0.05, **p<0.01, ***p<0.001, ****p<0.0001).

To determine the distribution of T cell subsets in HIV-infected aging patients who are successfully treated with long-term ART, we assessed 100 virologically controlled (<100 HIV RNA copies/mL) HIV-infected patients (HIV+) (median age 53 years and CD4 T cell counts 528 cells/mL) and compared with HIV-uninfected controls (UNC) (median age 53 years and CD4 T cell counts 958 cells/mL). Overall there was a 1.6-fold reduction in CD4^+^ T cell frequencies (HIV+: 28.76%, UNC: 47.41%) and a 2-fold increase in the frequencies of CD8^+^ T cells (HIV+: 35.29%, UNC: 16.95%) in HIV+ than UNC (Fig 1A). In addition, the frequencies of non-CD3^+^ lymphocyte populations, such as B cells and NK cells were similar in HIV+ and UNC, 35.95% and 35.63%, respectively (Fig 1A). We determined whether the CD8^+^ T cell frequencies were modulated with increasing CD4 T cell counts in HIV+ and found a decrease (p<0.0001) in the median frequencies of CD8^+^ T cells with increasing CD4 T cell counts in HIV+ compared with no change in the UNC (Fig 1B). Furthermore, there was a difference (p = 0.0021) between the slopes of HIV+ and UNC, as the slope for HIV+ showed a significant decline.

We next assessed T cell subsets, including naïve (N), central memory (CM) and effector memory (EM) in both our HIV+ and UNC. As expected, we saw a decrease in the median frequencies of both naïve CD4^+^ (20.98%) (p<0.0001) and CD8^+^ T cells (9.81%) (p<0.0001) in HIV+ compared with UNC (CD4^+^: 30.23%, CD8^+^:18.84%) (Fig 1C and 1D). However, the median frequencies of CD4^+^ CM T cells were increased (p = 0.01) (32.34%) (Fig 1C) and CD8^+^ CM T cells were slightly decreased (11.48%) (Fig 1D) in HIV+ compared with UNC (CD4^+^: 28.62%, CD8^+^: 12.78%). Also, the frequencies of EM CD4^+^ (7.38%) (p<0.0001) and CD8^+^ (51.57%) (p<0.0001) T cells showed increases in HIV+ compared with UNC (CD4^+^: 2.5%, CD8^+^:32.38%) (Fig 1C and 1D).

### Distribution of CD4^+^ and CD8^+^ T cells expressing CD28, CD27 and CD57 markers in virologically controlled HIV-infected aging patients on ART

**Fig 2:**
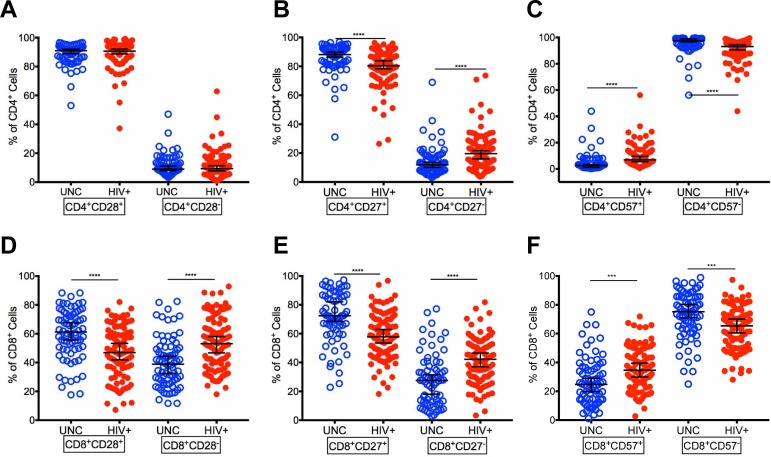
Distribution of CD4^+^ and CD8^+^ T cell phenotypes based on expression of CD28, CD27 and CD57 in HIV-infected patients with controlled viremia on ART. Frequencies of CD28^+^/CD28^-^ cells (A), CD27^+^/CD27^-^ cells (B), CD57^+^/CD57^-^ cells (C), within the CD4^+^ T cell population in HIV+ and UNC. Frequencies of CD28^+^/CD28^-^ cells (D), CD27^+^/CD27^-^ cells (E), CD57^+^/CD57^-^ cells (F) within the CD8^+^ T cell population in HIV+ and UNC. (n = 100 HIV^+^, n = 75 UNC) (*p<0.05, **p<0.01, ***p<0.001, ****p<0.0001).

First, we analyzed the costimulatory, CD28 and CD27, and terminal differentiation, CD57, markers on CD4^+^ and CD8^+^ T cells in our virologically controlled HIV+ and compared with UNC. The median frequencies of CD4^+^ T cells expressing CD28 (HIV+: 90.6%, UNC: 91%) and lacking CD28 expression (CD28^-^) (HIV+: 9.37%, UNC: 8.97%) were similar in HIV+ and UNC (Fig 2A). However, the CD8^+^ T cell population showed a decrease in the frequencies of CD8^+^CD28^+^ T cells (p<0.0001) (HIV+: 46.9%, UNC: 61.2%) and increase in CD8^+^CD28^-^ (p<0.0001) (HIV+: 53.1%, UNC: 38.8%) expression in HIV+ than UNC (Fig 2D). We also examined costimulatory molecule, CD27, important in T cell maintenance and memory [26], which showed a decrease in both the frequencies of CD4^+^CD27^+^ (p<0.0001) (Fig 2B) (HIV+: 80.4%, UNC: 88.1%) and CD8^+^CD27^+^ T cells (p<0.0001) (Fig 2E) (HIV+: 57.8%, UNC: 72.4%) in HIV+ than UNC. In addition, there was an increase in the frequencies of both CD4^+^CD27^-^ (p<0.0001) (Fig 2B) (HIV+:19.6%, UNC: 11.9%) and CD8^+^CD27^-^ (p<0.0001) (Fig 2E) (HIV+: 42.2%, UNC: 27.6%) T cells in HIV+ than UNC. Furthermore, terminally differentiated marker CD57 was evaluated on both CD4^+^ and CD8^+^ T cells that showed an increase in the frequencies of CD4^+^CD57^+^ (p<0.0001) (HIV+: 6.88%, UNC: 2.52%) and CD8^+^CD57^+^ (p<0.0001) (HIV+: 34.6%, UNC: 24.8%) in HIV+ than UNC. Also, there was a decrease in the frequencies of CD4^+^CD57^-^ (p<0.0001) (HIV+: 93.1%, UNC: 97.5%) (Fig 2C) and CD8^+^CD57^-^ T cells (p = 0.0001) (HIV+: 65.4%, UNC: 75.2%) (Fig 2F) in HIV+ compared with UNC. Our data show that the median frequencies of both CD4^+^ and CD8^+^ CD57^+^ T cells (Fig 2C and 2F) in our HIV+ cohort with controlled viremia is considerably lower than HIV+ patients with uncontrolled viremia reported elsewhere [1, 13].

Further comparison shows that the median frequencies of CD28^+^, CD27^+^ and CD57^-^ were higher than CD28^-^, CD27^-^ and CD57^+^ CD4/CD8 T cells both in HIV+ and UNC, except HIV+ had higher frequencies of CD8^+^CD28^-^ than CD8^+^CD28^+^ T cells likely due to elevated levels of effector memory CD8^+^ T cells (Fig 2A and 2D). In addition, the slightly higher median frequencies of CD28^-^, CD27^-^ and CD57^+^ in both CD4^+^ and CD8^+^ T cells in HIV+ than UNC could be due to lower median CD4 T cell counts of 528 in HIV+ versus 958 in UNC.

### Reduction of CD28^-^, CD27^-^ and CD57^+^ CD4^+^ and CD8^+^ T cells in virologically controlled HIV-infected aging patients on ART with increasing CD4 T cell counts

**Fig 3:**
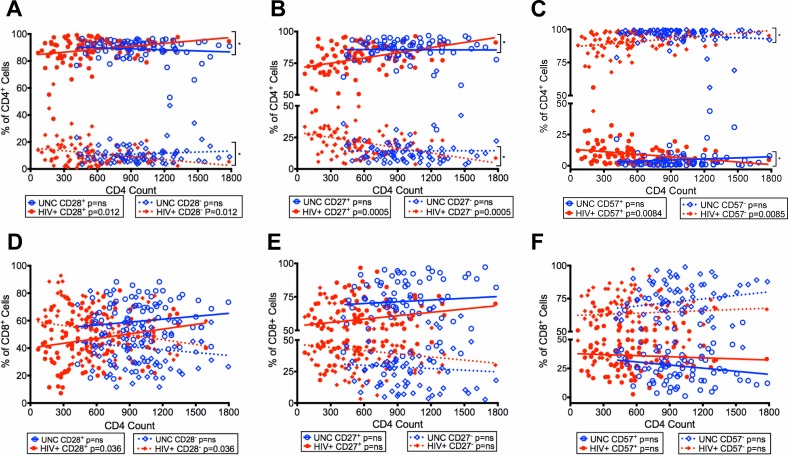
Reduced frequencies of CD28^-^, CD27^-^ and CD57^+^ T cells in HIV-infected patients with controlled viremia on ART with increasing CD4 T cell counts. Frequencies of CD28^+^/CD28^-^ cells (A), CD27^+^/CD27^-^ cells (B), CD57^+^/CD57^-^ cells (C) within the CD4^+^ T cell population plotted with increasing CD4 T cell counts in HIV+ and UNC. Frequencies of CD28^+^/CD28^-^ cells (D), CD27^+^/CD27^-^ cells (E), CD57^+^/CD57^-^ cells (F) within the CD8^+^ T cell population plotted with increasing CD4 T cell counts in HIV+ and UNC. (n = 100 HIV^+^, n = 75 UNC) (*p<0.05, **p<0.01, ***p<0.001, ****p<0.0001).

Next, we evaluated the expression of CD28, CD27, and CD57 on CD4^+^ and CD8^+^ T cells with increasing CD4 T cell counts in our HIV+. The CD4^+^ and CD8^+^ T cells of HIV+ showed an increase in the median frequencies of CD4^+^CD28^+^ (p = 0.012) and CD8^+^CD28^+^ T cells (p = 0.036) paired with a decline in the frequencies of CD4^+^CD28^-^ (p = 0.012) and CD8^+^CD28^-^ (p = 0.036) T cell with increasing CD4 T cell counts in HIV+ compared with no change in UNC (Fig 3A and 3D). There was a difference (p = 0.018) in the rate of change for CD4^+^CD28^+^ T cells between HIV+ and UNC (HIV+: positive, UNC: negative slope) and CD4^+^CD28^-^ (HIV+: negative, UNC: positive slope) (Fig 3A). We also found an increase in the frequencies of CD4^+^CD27^+^ (p = 0.0005) and CD8^+^CD27^+^, and a decrease of CD4^+^CD27^-^ (p = 0.0005) and CD8^+^CD27^-^ T cells in HIV+ with increasing CD4 T cell counts (Fig 3B and 3E) as compared to no change in UNC. Also, there was a difference (p = 0.0139) in the rate of change of CD27 between HIV+ and UNC for CD4^+^CD27^+^ T cells (HIV+; positive, UNC negative slope) and CD4^+^CD27^-^ (HIV+: negative, UNC: positive slope) (Fig 3B).

We examined the expression of CD57 and found that there was a reduction in the frequencies of CD4^+^CD57^+^ (p = 0.0084) and CD8^+^CD57^+^ T cells with increasing CD4 T cell counts in HIV+ compared with no change in UNC (Fig 3C and 3F). We also observed a difference in the rate of change (p = 0.0111) for CD57 expression on CD4^+^ T cells between HIV+ and UNC (CD57^+^: decrease in HIV+, increase in UNC; CD57^-^: increase in HIV+, decrease in UNC) (Fig 3C). These combined data on CD28, CD27 and CD57 demonstrate that there was an increase in the frequencies of CD28^+^, CD27^+^ and CD57^-^ CD4^+^ and CD8^+^ T cells with increasing CD4 T cell counts in our HIV+ with controlled viremia, suggesting improvement in the expression of costimulatory molecule CD28 and 27 and reduction in the terminal differentiation marker CD57 on T cells.

### Reduction of terminally differentiated, CD28^-^CD57^+^ CD4^+^ and CD8^+^ T cells in virologically controlled HIV-infected aging patients on ART with increasing CD4 T cell counts

Earlier studies have shown an inverse relationship in the expression of CD28 and CD57 on CD8^+^ T cells (CD8^+^CD28^-^CD57^+^) as markers of terminal differentiation and referred as premature aging of T cells in HIV-infected patients with uncontrolled viremia resulting in a rapid HIV disease progression [1, 2]. Cao et.al. [1] further defined three categories of memory CD8^+^ T cell subsets in uncontrolled viremic HIV+ patients, including early (CD8^+^CD28^+^CD57^-^), intermediate (CD8^+^CD28^-^CD57^-^), and late stage terminally differentiated (CD8^+^CD28^-^CD57^+^) T cells. We characterized these memory subsets of differentiated T cells in both CD4^+^ and CD8^+^ T cell populations in our HIV+ patients that have achieved controlled viremia and restored CD4 T cell counts due to long-term ART.

**Fig 4:**
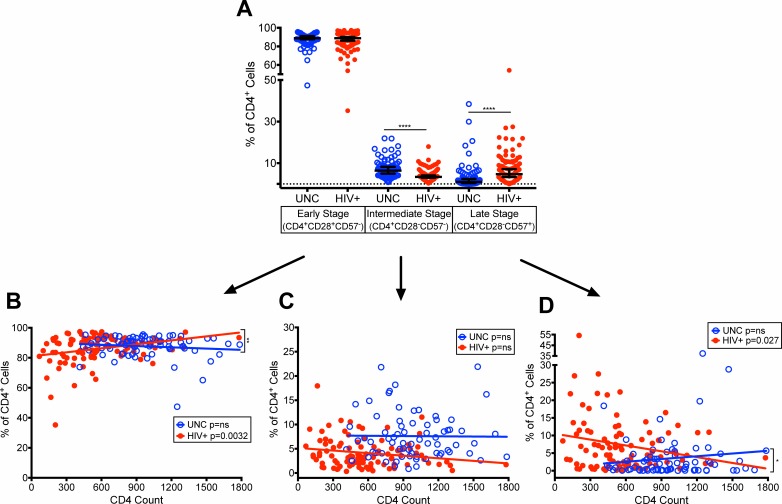
Characterization of early, intermediate, and late stage terminally differentiated CD4^+^ T cell subsets in HIV-infected patients with controlled viremia on ART. Frequencies of CD4^+^ T cell subsets defined by expression patterns of CD28 and CD57 in HIV+ patients and uninfected individuals (A). Frequencies of (B) early stage (CD28^+^CD57^-^), (C) intermediate stage (CD28^-^CD57^-^), and (D) late stage (CD28^-^CD57^+^) cells within the CD4^+^ T cell population plotted with increasing CD4 T cell counts in HIV+ and UNC. (n = 100 HIV^+^, n = 75 UNC) (*p<0.05, **p<0.01, ***p<0.001, ****p<0.0001).

The median frequencies of early stage CD4^+^CD28^+^CD57^-^ T cells showed no change between HIV+ (88.86%) and UNC (88.82%), a decrease in intermediate stage CD4^+^CD28^-^CD57^-^ T cells (p<0.0001) in HIV+ (3.41%) than UNC (6.42%) and an increase in late stage terminally differentiated CD4^+^CD28^-^CD57^+^ T cells (p<0.0001) in HIV+ (4.71%) than UNC (1.00%) (Fig 4A). It is important to note that the frequencies of intermediate and late stage terminally differentiated CD4^+^ T cells were much lower than early stage cells likely due to controlled viremia. Also, the higher frequencies of late stage CD4^+^ T cells in HIV+ than UNC could be due to the difference in the median CD4 T cell counts between the two cohorts. However, we observed significant improvements in these three stages of differentiated CD4^+^ T cells with increasing CD4 T cell counts in our HIV+ with controlled viremia due to ART (Fig 4B, 4C and 4D) compared with previously described HIV-infected patients with uncontrolled viremia [1–3, 25]. Our HIV+ cohort showed an increase in the median frequencies of early stage CD4^+^CD28^+^CD57^-^ T cells (p = 0.0032) (Fig 4B), a slight decline in intermediate stage CD4^+^CD28^-^CD57^-^ T cells (Fig 4C) and a significant decline (p = 0.027) in the late stage CD4^+^CD28^-^CD57^+^ terminally differentiated T cells (Fig 4D) with increasing CD4 T cell counts compared with no change in the UNC. This reduction in late stage terminally differentiated CD4^+^CD28^-^CD57^+^ T cells, is suggestive of improvement in exhaustion/senescence of CD4^+^ T cells, as the CD4 T cell counts increased in HIV+. In addition, a significant difference was seen in the rate of change for the CD4^+^ early stage T cells (p = 0.0071) (HIV+: increase; UNC: decrease) (Fig 4B), and late stage T cells (p = 0.0257) (HIV+: significantly decrease; UNC slightly decrease) (Fig 4D) with increasing CD4 T cell counts.

**Fig 5:**
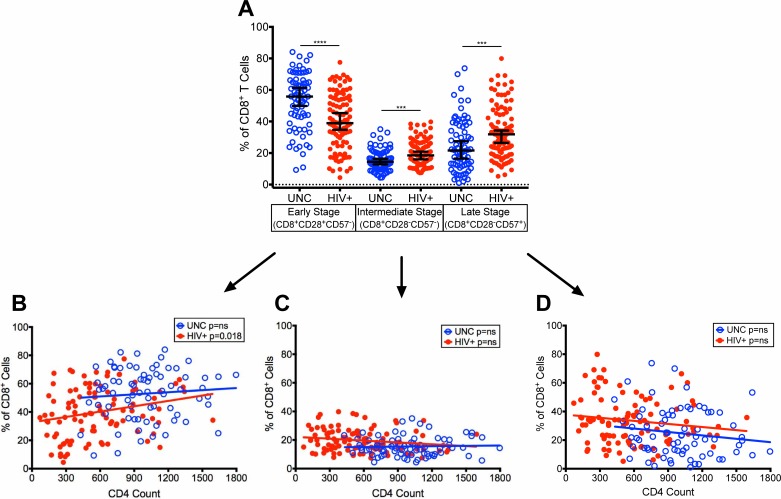
Characterization of early, intermediate, and late stage terminally differentiated CD8^+^ T cell subsets in HIV-infected patients with controlled viremia on ART. Frequencies of CD8^+^ T cell subsets defined by expression patterns of CD28 and CD57 in HIV+ and UNC (A). Frequencies of early stage (CD28^+^CD57^-^) (B), intermediate stage (CD28^-^CD57^-^) (C), and late stage (CD28^-^CD57^+^) (D) cells within the CD8^+^ T cell population plotted with increasing CD4 T cell counts in HIV+ and UNC. (n = 100 HIV^+^, n = 75 UNC) (*p<0.05, **p<0.01, ***p<0.001, ****p<0.0001).

In the analysis of the CD8^+^ T cell population, there was a decrease (p<0.0001) in the median frequencies of the early stage CD8^+^CD28^+^CD57^-^ T cells in HIV+ (38.96%) than UNC (55.83%) (Fig 5A), an increase in both intermediate (p = 0.0003) (CD28^-^CD57^-^) in HIV+ (18.46%) than UNC (14.46%) and late stage (CD28^-^CD57^+^) (p = 0.0004) in HIV+ (31.87%) than UNC (21.59%) CD8^+^ T cells (Fig 5A). Like CD4^+^ T cells, the median frequencies of intermediate and late stage terminally differentiated CD8^+^ T cells were also lower than early stage cells, and that the higher frequencies of these cells in HIV+ than UNC could be attributed to a lower median CD4 T cell counts in HIV+ than UNC. However, when we evaluated these three memory CD8^+^ T cell subsets with increasing CD4 T cell counts, we found an increase in the frequencies of early stage CD8^+^CD28^+^CD57^-^ T cells (p = 0.018) in HIV+ and no change in UNC (Fig 5B) with increasing CD4 T cell counts. While there were no significant changes in the frequencies of intermediate stage CD8^+^CD28^-^CD57^-^ T cells, we observed a declining rate in these HIV+ cells with increasing CD4 T cell counts compared with an increasing rate in UNC (Fig 5C). Furthermore, there was a decline in the frequencies of late stage terminally differentiated CD8^+^CD28^-^CD57^+^ T cells with increasing CD4 T cell counts in HIV+ (Fig 5D). These data suggest that there was a reduction in terminally differentiated CD8^+^ T cells with increasing CD4 T cell counts in HIV+ patients with controlled viremia due to ART, suggestive of improvement in exhaustion/senescence of CD8^+^ T cells.

### Improvement in naïve to memory T cell ratios in virologically controlled HIV-infected aging patients on ART with increasing CD4 T cell counts

**Fig 6:**
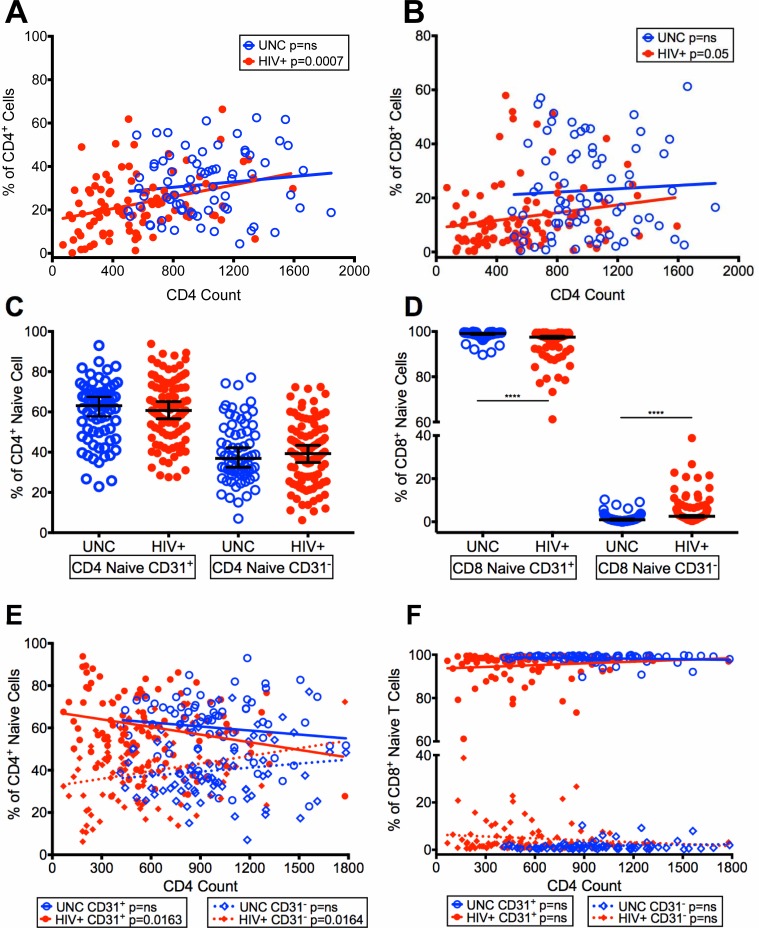
Recovery of CD4^+^ and CD8^+^ naïve to memory T cell ratios in HIV-infected patients with controlled viremia on ART. Frequencies of naïve (N, CD28^Int^CD95^Lo^CD45RA^Hi^CCR7^Hi^) CD4^+^ T cells (A), and CD8^+^ T cells (B) plotted with increasing CD4 T cell counts in HIV+ and UNC. Frequencies of CD31^+^ and CD31^-^ cells on naïve CD4^+^ T cells (C), and naïve CD8^+^ T cells (D) in HIV+ and UNC. Frequencies of CD31^+^ and CD31^-^ cells on naïve CD4^+^ T cells (E), and naïve CD8^+^ T cells (F) plotted with increasing CD4 T cell counts in HIV+ and UNC. (n = 100 HIV^+^, n = 75 UNC) (*p<0.05, **p<0.01, ***p<0.001, ****p<0.0001).

To further investigate the changes in the naïve CD4^+^ and CD8^+^ T cell populations, we analyzed these T cells with increasing CD4 T cell counts in our HIV+ with controlled viremia. There was a significant increase in the frequencies of CD4^+^ naïve (p = 0.0007) (Fig 6A) and CD8^+^ naïve T cells (p = 0.05) (Fig 6B) with increasing CD4 T cell counts in HIV+ compared with UNC, suggesting an improvement in naïve to memory T cell ratios. Next, we investigated whether the increased frequencies of naïve CD4^+^ and CD8^+^ T cells (Fig 6A and 6B) are from recent thymic emigrants (RTE, CD31^+^) or from those proliferating in the periphery (CD31^-^). Fig 6C and 6D show that the HIV+ had significantly higher median frequencies of CD4^+^ and CD8^+^ CD31^+^ naïve T cells (CD4^+^: 60.7%, CD8^+^: 97.5%) compared with CD4^+^ and CD8^+^ CD31^-^ naïve T cells (CD4^+^: 39.3%, CD8^+^: 2.54%), with similar distribution in UNC, suggesting that the increased naïve CD4^+^ and CD8^+^ T cells were RTE.

We also examined the frequencies of naïve CD4^+^ CD31^+/-^ T cells with increasing CD4 T cell counts and found that the CD4^+^CD31^+^ naïve T cells decreased (p = 0.0163) and CD4^+^ CD31^-^ naïve T cells increased (p = 0.0164) in HIV+ compared with UNC (Fig 6E). These data suggest that the CD4^+^ naïve T cell population in HIV+ patients that had lower CD4 T cell counts were RTE (CD31^+^); whereas those HIV+ patients that have achieved higher CD4 T cell counts (such as <1300) had increased CD31^-^ (Fig 6E). However, while no changes were observed in the frequencies of naïve CD8^+^CD31^+/-^ T cells in the HIV+ with increasing CD4 T cell counts (Fig 6F), most of naïve CD8^+^ T cells were CD31^+^ (RTE) and very little were CD31^-^.

### Evaluation of CD4^+^ T cell function, IL-2, IL-10 and IFN-γ production, in virologically controlled HIV-infected aging patients on ART with increasing CD4 T cell counts

**Fig 7:**
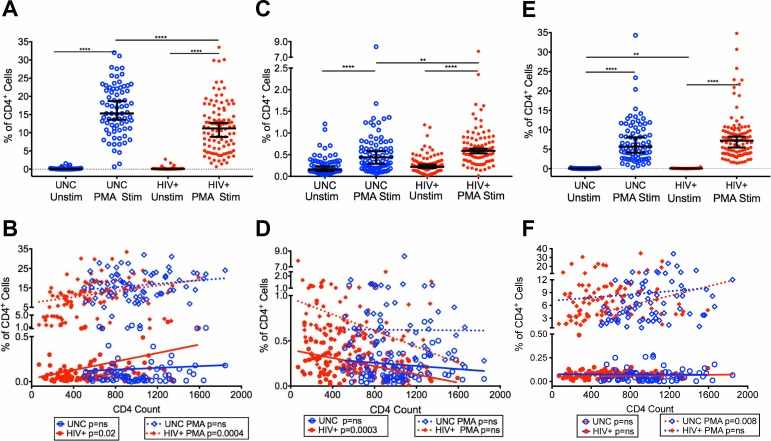
Evaluation of CD4^+^ T cell function, IL-2, IL-10 and IFN-γ production, in HIV-infected patients with controlled viremia on ART. Frequencies of unstimulated and PMA stimulated IL-2 (A), IL-10 (C) and IFN-γ (E) CD4^+^ T cells in HIV+ and UNC. Frequencies of unstimulated and PMA stimulated IL-2 (B) IL-10 (D), IFN-γ (F) CD4^+^ T cells plotted with increasing CD4 T cell counts in HIV+ and UNC. (n = 100 HIV^+^, n = 75 UNC) (*p<0.05, **p<0.01, ***p<0.001, ****p<0.0001).

To this point, the data presented above show a reduction in the levels of terminally differentiated T cells (Fig 5) and an improvement in the ratios of naïve to memory T cells (Fig 6) in our HIV+ patients that have controlled viremia and restored CD4 T cell counts due to ART. We further evaluated the capacity of the CD4^+^ T cells to produce cytokines, including IL-2, IL-10 and IFN-γ to demonstrate T cell function. First, we analyzed the unstimulated levels of intracellular IL-2 produced by CD4^+^ T cells and found that the median frequencies of CD4^+^IL-2 T cells were very low in both HIV+ (0.073%) and UNC (0.057%) (Fig 7A). However, upon stimulation with PMA both HIV+ and UNC showed an increase (p<0.0001) in the frequencies of CD4^+^IL-2 T cells (HIV+: 11.2%, UNC: 15.3%) compared with unstimulated cells (Fig 7A), but the frequencies of CD4^+^IL-2 T cells were significantly lower (p<0.0001) in HIV+ compared with UNC. We also found that the frequencies of both unstimulated and PMA stimulated CD4^+^IL-2^+^ T cells significantly increased (p = 0.0259 and p = 0.0004, respectively) with increasing CD4 T cell counts in HIV+ compared with UNC (Fig 7B), demonstrating an improvement in CD4^+^ T cells function in our HIV+.

Next, we determined the median frequencies of CD4^+^ T cells producing the anti-inflammatory cytokine, IL-10, in unstimulated cells and found that the HIV+ showed a slight increase compared with UNC, although the levels were very low (HIV+: 0.22%, UNC: 0.16%) (Fig 7C). However, the median frequencies of CD4^+^IL-10 T cells were significantly increased (p<0.0001) after PMA stimulation, including higher levels (p = 0.0062) in HIV+ than UNC (HIV+: 0.59%, UNC: 0.44%) (Fig 7C). The increased level of IL-10 in HIV+ is likely to counter the increased inflammatory process. Interestingly, we found that the frequencies of CD4^+^IL-10 T cells significantly declined (p = 0.0003) in unstimulated and PMA stimulated cells with increasing CD4 T cell counts in HIV+ compared with UNC (Fig 7D). We also assessed the capacity of the CD4^+^ T cells of HIV+ to produce the proinflammatory cytokine, IFN-γ. We found that the median frequencies of unstimulated CD4^+^IFN-γ T cells were higher (p = 0.0021) in HIV+ compared with UNC (HIV+: 0.069%, UNC: 0.051%) (Fig 7E). Moreover, both HIV+ and UNC showed an increase (p<0.0001) in the frequencies of CD4^+^IFN-γ T cells upon PMA stimulation, and the levels were higher in HIV+ (7.22%) compared with UNC (5.69%) (Fig 7E), suggesting some level of inflammation existed in our HIV+ with controlled viremia. We also found that the frequencies of unstimulated HIV+ CD4^+^IFN-γ T cells slightly decreased with increasing CD4 T cell counts compared with a slight increase in UNC (Fig 7F). Upon PMA stimulation, HIV+ patients showed a slight increase in the frequencies of CD4^+^IFN-γ T cells compared with a significant increase (p = 0.008) in UNC (Fig 7F).

## Discussion

Here we provide evidence for immunological reconstitution, including a reduction in terminally differentiated T cells, based on the frequencies of CD4^+^/CD8^+^ CD28^-^, CD27^-^, CD57^+^ and CD28^-^CD57^+^ T cells, an improvement in ratios of naïve to memory and function of T cells in HIV-infected patients with controlled viremia and restored CD4 T cell counts due to ART compared with increased frequencies of terminally differentiated T cells in previously studied untreated HIV-infected patients with uncontrolled viremia [1, 3]. More importantly, we saw a phenotypic shift from CD28^-^, CD27^-^, CD57^+^ and CD28^-^CD57^+^ to CD28^+^, CD27^+^, CD57^-^ and CD28^+^CD57^-^ T cells moving away from terminal differentiation [19, 20]. In addition, the increased naïve CD4^+^ T cells in our HIV-infected patients were due to CD4^+^CD31^+^ (RTE), and the CD4^+^ T cells showed improved functions by producing cytokines, including IL-2, IL-10 and IFN-γ. Taken together, these findings suggest that reduction in terminally differentiated T cells, an indication of reversal of exhaustion/senescence of T cells, and improvement in ratios of naïve to memory and function of T cells occurred in HIV-infected patients likely due to suppression of viremia and improvement of CD4 T cell counts due to long-term ART.

Following accumulation of CD28^-^ T cells during HIV infection, it has been reported that CD57^+^ terminally differentiated T cells begin to accumulate [3, 5, 25, 27] that have a reduced capacity to proliferate [1, 2]. Several studies have reported that HIV-infected patients that are either ART naïve or on short-term ART have reduced frequencies of early stage (CD28^+^CD57^-^) and increased frequencies of intermediate (CD28^-^CD57^-^) and late stage (CD28^-^CD57^+^) terminally differentiated CD4^+^ and CD8^+^ T cells compared with uninfected individuals [1, 2]. However, our study found that HIV-infected patients on long-term ART with controlled viremia and improved CD4 T cell counts have increased accumulation of early stage (CD28^+^CD57^-^), and decreased intermediate (CD28^-^CD57^-^) and late (CD28^-^CD57^+^) stage terminally differentiated CD4^+^ and CD8^+^ T cells (Figs 4 and 5). These data demonstrate that there was a reduction in terminal differentiation of T cells suggesting likely recovery from exhaustion/senescence.

An important question remains whether the mechanism of reduction in terminally differentiated T cells is due to increases in the naïve T cell pool or recruitment of new memory T cells. Unlike previous studies that used two markers CD45RA and CD27 for differentiation of naïve T cells [1, 28], we expanded on these markers to include CD28, CD45RA, CD95, and CCR7, for a more precise examination of the naïve T cell population. This expansion eliminated the possibility of contamination of our naïve T cell results with a population of effector T cells that express the phenotype CD3^+^CD4^+^/CD8^+^CD45RA^+^CD27^+^ [28]. Numerous studies have shown that an increased accumulation of terminally differentiated T cells is due to a decline of both CD4^+^ and CD8^+^ naïve T cells and referred as accelerated senescence of T cells in HIV-infected patients with uncontrolled viremia [1, 3, 5, 25]. However, the reduction in terminally differentiated T cells as seen in our HIV+ patients with controlled viremia due to ART is likely due to increases in CD4^+^ and CD8^+^ naïve T cells and the improved naïve to memory T cell ratios with increasing CD4 T cell counts in HIV+ patients (Fig 1). As thymic output decreases there is a loss in TCR repertoire and an accumulation of CD31^-^ naïve T cells in the aging process [4, 27] and this is accelerated in HIV-infected patients with uncontrolled viremia [3, 5, 25]. Our study provides the mechanism behind the increased naïve CD4^+^ and CD8^+^ T cells, which are due to recent thymic emigrants (RTEs), the CD31^+^ T cells. Our data suggest a recovery of the TCR repertoire and reduction in previously reported terminal differentiation of T cells [1, 3], indicating improvement in exhaustion/immunosenescence.

Although our study showed phenotypic recovery of the reconstituted CD4^+^ T cells, we further strengthened our data by demonstrating that these CD4^+^ T cells produce important cytokines, including IL-2, IL-10 and IFN-γ that are critical for T cell functions [29]. Since IL-2, an important cytokine for CD4^+^ T cell functionality [30], expression is downregulated during HIV infection, its increased production in our HIV+ patients supports improved T cell function. IL-10, an anti-inflammatory and suppressor of Th1 cytokine [29, 31], is significantly upregulated in HIV infection to counter inflammation and is correlated with HIV disease progression [31]. Our data shows that while IL-10 was produced at higher levels with lower CD4 T cell counts in HIV+, the IL-10 levels decreased with increasing CD4 T cell counts in HIV+ (Fig 7D). Lastly, IFN-γ made by CD4^+^ T cells that enhances CD8^+^ T and NK cells activities against HIV-infected cells [29, 30], but the stimulation of these cells was shown to be diminished in HIV infection due to depletion of CD4^+^ T cells. The data presented here show that with increasing CD4 T cell counts in HIV-infected patients, the levels of IFN-γ in CD4^+^ T cells was significantly improved (Fig 7E and 7F). Collectively, our data on production of these cytokines by CD4^+^ T cells of HIV+ patients suggest improved T cell functions with controlled viremia and improved CD4 T cell counts.

In conclusion, our findings suggest that virologically controlled HIV+ patients receiving long-term ART with restored CD4 T cell counts can achieve immune reconstitution both phenotypically and functionally compared with HIV+ patients with uncontrolled viremia [1, 3]. On a cellular level the reconstitution of the naïve CD4^+^ and CD8^+^ T cells leads to a reduction in the populations of terminally differentiated CD4^+^ and CD8^+^ T cells, suggestive of improved exhaustion/senescence of the T cells, as shown in our study. Nevertheless, future studies should aim to investigate the functional capabilities of each subset of T cells and functional recovery of exhausted/senescent T cells with more defined and elaborated markers [32]. Furthermore, while the number of HIV+ patients (n = 100) and uninfected controls (n = 75) included has increased the power of our study that provided statistically significant data, one of the limitations is that the blood samples used for analysis were only from a single time point. This limitation can be circumvented by including longitudinal samples over an extended period to determine the changes in the phenotypes and functions of T cells from these virologically controlled HIV-infected aging patients. Our study showed recovery from terminal differentiation of T cells with improved T cell functions, which may have implications on developing new modalities for HIV treatment, and in controlling acute and chronic infections and improving efficacy of vaccinations [33] in HIV+ patients with controlled viremia.
